# Feasibility of Adjunct Therapy with a Robotic Hand Orthosis after Botulinum Toxin Injections in Persons with Spasticity: A Pilot Study

**DOI:** 10.3390/toxins16080346

**Published:** 2024-08-08

**Authors:** Raffaele Ranzani, Margherita Razzoli, Pierre Sanson, Jaeyong Song, Salvatore Galati, Carlo Ferrarese, Olivier Lambercy, Alain Kaelin-Lang, Roger Gassert

**Affiliations:** 1 Rehabilitation Engineering Laboratory, Department of Health Sciences and Technology, ETH Zurich, Gloriastrasse 37/39, 8092 Zurich, Switzerland; razzolimargherita@gmail.com (M.R.); pierresanson59@gmail.com (P.S.); jay.song@hest.ethz.ch (J.S.); olivier.lambercy@hest.ethz.ch (O.L.); roger.gassert@hest.ethz.ch (R.G.); 2 School of Medicine and Surgery and Milan Center for Neuroscience (NeuroMi), University of Milano-Bicocca, Piazza dell’Ateneo Nuovo 1, 20126 Milan, Italy; carlo.ferrarese@unimib.it; 3 Cereneo, Center for Neurology and Rehabilitation, Seestrasse 18, 6354 Vitznau, Switzerland; 4 Faculty of Biomedical Sciences, Università della Svizzera Italiana, 6962 Lugano, Switzerland; salvatore.galati@eoc.ch (S.G.); alain.kaelin@eoc.ch (A.K.-L.); 5 Neurology Department, Neurocenter of Southern Switzerland, Ente Ospedaliero Cantonale, 6900 Lugano, Switzerland; 6 Department of Neurology, Inselspital, Bern University Hospital, University of Bern, 3010 Bern, Switzerland

**Keywords:** botulinum toxin, robotic hand orthosis, exoskeleton, hand, grasp function, robot-assisted rehabilitation, stroke, neurorehabilitation, spasticity treatment, muscle tone

## Abstract

Upper-limb spasticity, frequent after central nervous system lesions, is typically treated with botulinum neurotoxin type A (BoNT-A) injections to reduce muscle tone and increase range of motion. However, performing adjunct physical therapy post-BoNT-A can be challenging due to residual weakness or spasticity. This study evaluates the feasibility of hand therapy using a robotic hand orthosis (RELab tenoexo) with a mobile phone application as an adjunct to BoNT-A injections. Five chronic spastic patients participated in a two-session pilot study. Functional (Box and Block Test (BBT), Action Research Arm Test (ARAT)), and muscle tone (Modified Ashworth Scale (MAS)) assessments were conducted to assess functional abilities and impairment, along with usability evaluations. In the first session, subjects received BoNT-A injections, and then they performed a simulated unsupervised therapy session with the RELab tenoexo in a second session a month later. Results showed that BoNT-A reduced muscle tone (from 12.2 to 7.4 MAS points). The addition of RELab tenoexo therapy was safe, led to functional improvements in four subjects (two-cube increase in BBT as well as 2.8 points in grasp and 1.3 points in grip on ARAT). Usability results indicate that, with minor improvements, adjunct RELab tenoexo therapy could enhance therapy doses and, potentially, long-term outcomes.

## 1. Introduction

Spasticity is a common disorder following central nervous system (CNS) disorders, such as stroke, spinal cord injury (SCI), and multiple sclerosis. Traditionally, spasticity is defined as “a motor disorder characterized by a velocity-dependent increase in tonic stretch reflexes (‘muscle tone’) with exaggerated tendon jerks resulting from hyperexcitability of the stretch reflex as one component of the upper motor neuron syndrome” [1]. A more clinically oriented definition of spasticity is “disordered sensorimotor control, resulting from an upper motor neuron lesion, presenting an intermittent or sustained involuntary activation of muscles” [2]. Chronic spasticity is highly frequent, with an incidence ranging from 17–42.6% after stroke [3], 31% after SCI [4,5], 19–42% after traumatic brain injury [6], 61–76.9% in cerebral palsy, and 84% in multiple sclerosis [7,8]. Unlike lower-limb spasticity, which can sometimes aid in walking and standing, upper-limb and hand spasticity severely compromises functional independence and quality of life [9,10]. Uncontrolled long-term spasticity can lead to abnormal joint positioning, contractures, pain, and challenges with daily hygiene [10]. It impairs the ability to initiate or terminate functional grips or generate sustained grasp force due to the intrinsic weakness of spastic muscles [11,12,13].

Effective treatments for upper-limb spasticity are essential to enhance patients’ functional independence and quality of life, thus reducing the burden on caregivers and society [14]. Various treatment options are available (e.g., pharmacological treatments, physical therapy, orthoses, transcutaneous electrical stimulation, and surgery) and are often undertaken simultaneously [15,16]. Botulinum neurotoxin type A (BoNT-A) intramuscular injections are one of the most established treatment methods. Within seven to 14 days post-injection, BoNT-A produces a muscle tone reduction, which peaks at three to four weeks and lasts on average for three months [17]. This period of reduced muscle tone, partial analgesia, increased mobility, and increased range of motion could facilitate intensive physical therapy, potentially leading to improved functional outcomes [18,19,20,21]. However, due to residual spasticity, contractures, and BoNT-A-induced weakness, patients frequently require external support to be able to perform physical therapy [13,22]. This poses a challenge, since most chronic patients are outpatients with difficulties coordinating supervised physical therapy with BoNT-A injections [23,24].

Robotic technologies can help patients perform high-dose quality therapy without therapist support [25,26]. Previous studies have shown that combining BoNT-A injections with robot-assisted therapy improves outcomes in spasticity treatment [27,28,29]. Robotic hand orthoses (RHO) offer a portable and effective solution to enable unsupervised therapy (e.g., at the patient’s home), though their use in spastic patients faces challenges such as donning, tailoring size fit, and maintaining a compact size while achieving forces that are high enough to open a spastic hand without risking an injury [23,24].

This paper investigates the safety and feasibility of adjunct unsupervised therapy with a robotic hand orthosis after BoNT-A injections in a pilot study with subjects suffering from upper-limb spasticity due to CNS lesions. The RELab tenoexo was selected as the ideal RHO for its wearability, weight, compact design, and force range [30]. A new mobile phone application was developed to allow performing unsupervised therapy exercises with the RHO. The primary outcome of the study was to evaluate the safety (i.e., adverse events, situations at risk) and feasibility (i.e., performance checklist recording which items/tasks the subject could or could not perform without supervision) of adjunct BoNT-A injections and (simulated) unsupervised therapy with the RELab tenoexo system (i.e., RHO and mobile phone application). This serves as a first proof-of-concept for using this approach in real-world scenarios before evaluating its efficacy in a larger clinical trial. The study also has three secondary objectives. First, we perform a preliminary evaluation of whether this therapy approach could have immediate positive effects on upper-limb and hand function in a short therapy time. Second, changes in muscle tone related to the BoNT-A injections are accurately assessed with clinical and robotic assessments, and the relationship between these assessments is established. Third, the usability of the RELab tenoexo system is evaluated for adjunct use after BoNT-A injections in the given population. This work will help identify possible design improvements that are needed before a larger clinical trial can be performed. We hypothesize that the adjunct BoNT-A injections and RELab tenoexo therapy approach will show positive feasibility results and that BoNT-A injections will reduce muscle tone without direct functional benefits, which will instead arise through a combination with the RELab tenoexo.

## 2. Results

### 2.1. Study Participants

Between February and July 2023, six consecutive subjects with CNS lesions were screened to participate in the study. One subject was excluded due to finger contractures and a Modified Ashworth Scale (MAS) score of four. Two out of the five subjects included in the study (i.e., subjects two and three) could not perform the simulated unsupervised therapy session at S1 (i.e., second and last session, four to six weeks after BoNT-A injections), due to technical problems with the RHO (i.e., mechanical failure of the actuation unit). However, all the available data of the five subjects included in the study were analyzed. The baseline characteristics of the subjects are summarized in Table 1. Subjects had an average age of 59.5 (13.7) (mean (std)) years and experienced a CNS lesion 11.4 (8.2) years prior. CNS lesions included two ischemic strokes (one left and one right hemisphere) of the middle cerebral artery, one ischemic stroke of the left anterior and middle cerebral arteries, one occlusive dissection of the left internal carotid artery, and one cerebellar syndrome with sensorimotor hemisyndrome post-right vestibular Schwannoma resection. The subjects had multiple comorbidities or complications related to the CNS lesions, including lower-limb spasticity (2), seizures (2), permanent use of a wheelchair (1), depression (1), dystonia (1), ataxia (1), aphasia (1), as well as deep-vein thrombosis and pulmonary embolism (1). All subjects were already undergoing BoNT-A injection treatments, with the last injection on average 14.8 (1.3) weeks before the start of the study and a first session in which they were first exposed to the RHO and received BoNT-A injections (session S0).

### 2.2. Safety and Feasibility

No serious adverse events related to the intervention (i.e., use of the RELab tenoexo system and BoNT-A injections) or events that could compromise the safety of the user were observed during the study. Two subjects reported temporary pressure marks after removing the hand module.

**Figure 1:**
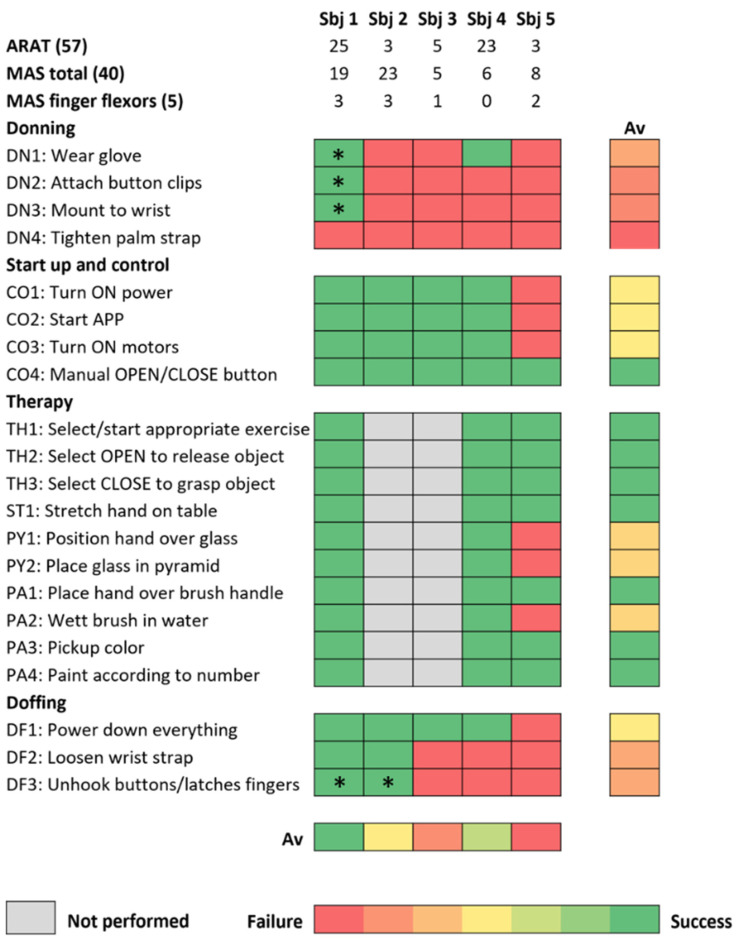
Checklist results represented as a heatmap. The results averaged over the subjects, and items are presented on the right and at the bottom of the heat map, respectively. The items “DN”, “CO”, and “DF” were evaluated during the assessments at S0 and S1, the other items only at S1. The tasks are ranked successful (1, green, i.e., no problem/issue in item completion without external intervention) or failed (0, red, i.e., failure and/or external intervention required to solve the item). Intermediate nuances between green and red are average values (Av) between 0 and 1 over subjects and items, respectively. DN: donning; CO: start-up and control; TH: all therapy exercises; ST: Stretching; PY: Pyramid; PA: Painting; *: item that could not be performed without external intervention before BoNT-A injections (S0) but could be performed after BoNT-A injections (S1).

**Figure 2:**
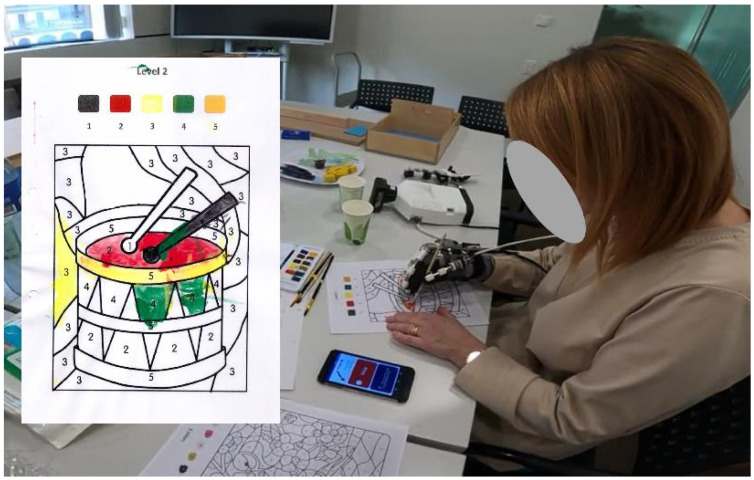
Subject one performing the painting exercise (difficulty level 2) and the resulting sheet after 10 min of exercise.

Subjects required external supervision or assistance for 80.0 (32.6)% of the checklist items related to donning the RELab tenoexo, 15.0 (33.5)% of the items related to starting up and controlling the RHO through the mobile phone application, and 46.7 (44.7)% of the items related to doffing the hand module (Figure 1). The subjects’ performance in the checklist items did not vary from S0 to S1, except for subjects one and two who initially had a high MAS in the fingers at S0 and benefited more from the BoNT-A injections. After BoNT-A injections at S1, subject one could wear the glove and attach the button clips and the wrist strap and, as subject two, could unlock the finger clips/straps and remove the glove at the end of the session. Only subjects one and four, who had an initially lower (but still moderate) upper-limb impairment in terms of the Action Research Arm Test (ARAT) score (25 and 23, respectively) and less-impaired shoulder function, could independently wear the glove and performed better, overall, in all checklist items. During the simulated unsupervised therapy session at S1, subjects still required external supervision or assistance for 10.0 (17.3)% of the checklist items. Only subject five, who had the lowest ARAT score (3), did not succeed in exercise tasks requiring lifting the arm (e.g., lifting a glass and placing it over the pyramid), which are part of exercises with higher task difficulty (i.e., Pyramid, Painting). The result of 10 min of the Painting exercise for subject one is shown in Figure 2.

### 2.3. Functional Benefit

**Figure 3:**
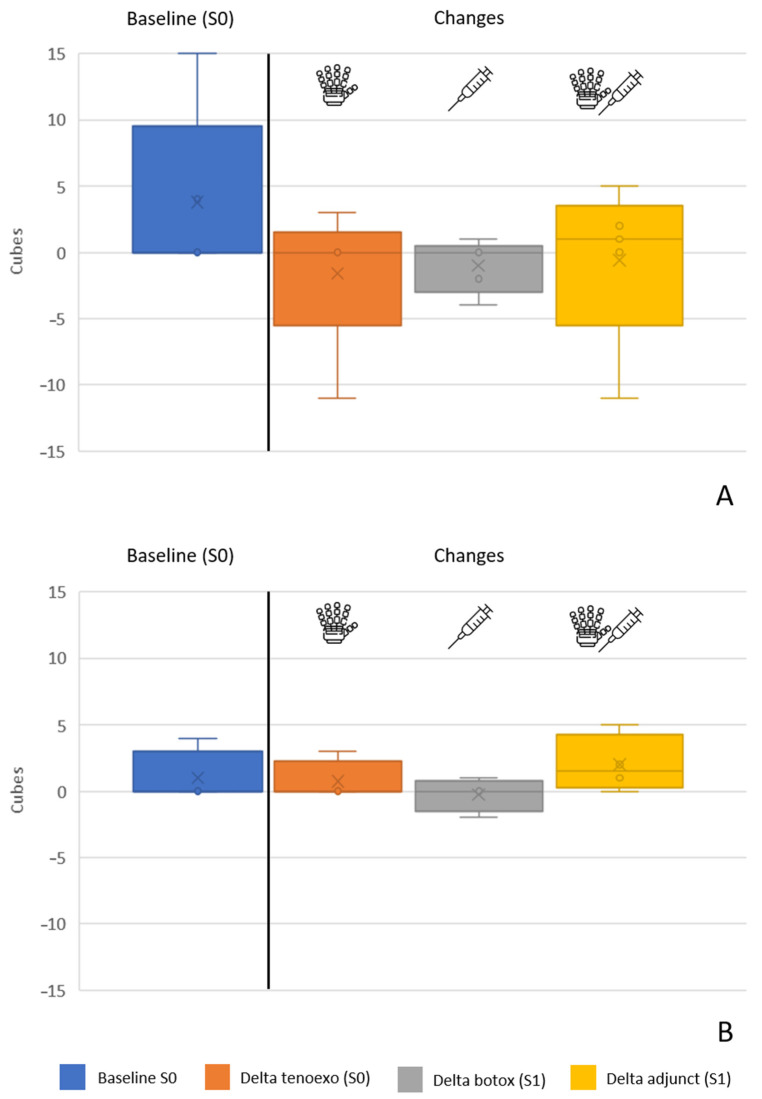
Box and Block Test box-plot results for all five subjects (**A**) and for the four subjects with muscle hypertonia in finger flexors (i.e., MAS ≥ 1) (**B**). On the left of the black line are the results at the baseline (S0), while on the right of the line are the changes in performance when using the RELab tenoexo before BoNT-A injections (S0), four to six weeks after BoNT-A injections without RELab tenoexo (S1), and adjunct BoNT-A injections and RELab tenoexo (S1). °: individual scores; ×: mean value.

The subjects had a baseline Box and Block Test (BBT) score of 3.8 (6.5) cubes over one minute, which decreased by 1.6 (5.4) cubes when using the RELab tenoexo at S0, 1.0 (2.0) cubes after BoNT-A injections at S1, and 0.6 (6.1) when using RELab tenoexo as adjunct to BoNT-A injections at S1 (Figure 3A). However, one subject, subject four, had a moderate ability level at the ARAT (i.e., 23) and a muscle tone level of zero at the MAS in the finger flexors. Given the absence of hypertonia in the finger flexors and the lack of distal impairment, this subject is considered an outlier with respect to the others. In fact, the use of BoNT-A injections and/or of the RHO, which can improve function in people with distal impairment, had the highest negative impact on performance compared to the baseline (i.e., a decrease of eleven and four blocks, respectively).

Excluding this subject from the analysis and considering only subjects with an increase in hand muscle tone, the BBT results vary considerably, as shown in Figure 3B. The other four subjects had a baseline BBT score of 1.0 (2.0), a mild decrease in performance after BoNT-A injections of 0.3 (1.3), and an increase in performance when using the RELab tenoexo or the combination of RELab tenoexo and BoNT-A injections of 0.8 (1.5) and 2 (2.2), respectively.

**Figure 4:**
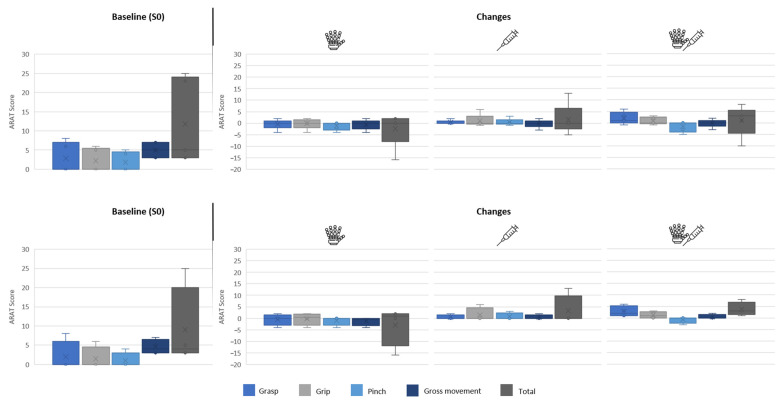
Action Research Arm Test (ARAT) results for all the subjects (**top**) and for the four subjects with muscle hypertonia in finger flexors (i.e., MAS ≥ 1) (**bottom**). On the left of the black line are the results at the baseline (S0), while on the right of the line are the changes in performance when using the RELab tenoexo (S0), BoNT-A injections (S1), and adjunct BoNT-A injections and RELab tenoexo (S1). °: individual scores; ×: mean value.

Subjects had a baseline ARAT score of 11.8 (11.2), as shown in Figure 4. Subjects two, three, and five showed very low upper-limb ability (i.e., three, five, and three, respectively), while subjects one and four had moderate upper-limb ability (i.e., 25 and 23, respectively). Compared to the baseline, the ARAT score decreased by 2.4 (7.7) points when using the RELab tenoexo at S0 then increased by 1.6 (6.7) points after BoNT-A injections at S1 and by 1.0 (6.7) points when using the RELab tenoexo as an adjunct to BoNT-A injections at S1.

As with the BBT results, subject four, who did not have hypertonia in the finger flexors, had more negative results when using the hand orthosis compared to the other four subjects. They had a baseline ARAT score of 9.0 (10.7), a mild decrease of 3.0 (8.7) in performance when using the RELab tenoexo, and a greater increase in performance after BoNT-A injections or through the combination of RELab tenoexo and BoNT-A injections of 3.3 (6.5) and 3.8 (3.0), respectively. Considering the ARAT subscores in these four subjects, grasp and grip function decreased, respectively, by 0.5 (2.5) and 0.3 (2.6) points with the RELab tenoexo, increased by 0.5 (1.0) and 1.5 (3.0) points, respectively, after BoNT-A, and increased by 2.8 (2.4) and 1.3 (1.5) points, respectively, with the adjunct use of BoNT-A and RELab tenoexo. The objects that showed greater functional benefit were the cubes of 2.5 and 5.0 cm side lengths and the stone sized 10.0 × 2.5 × 1.0 cm^3^.

### 2.4. Muscle Tone Assessment

**Figure 5:**
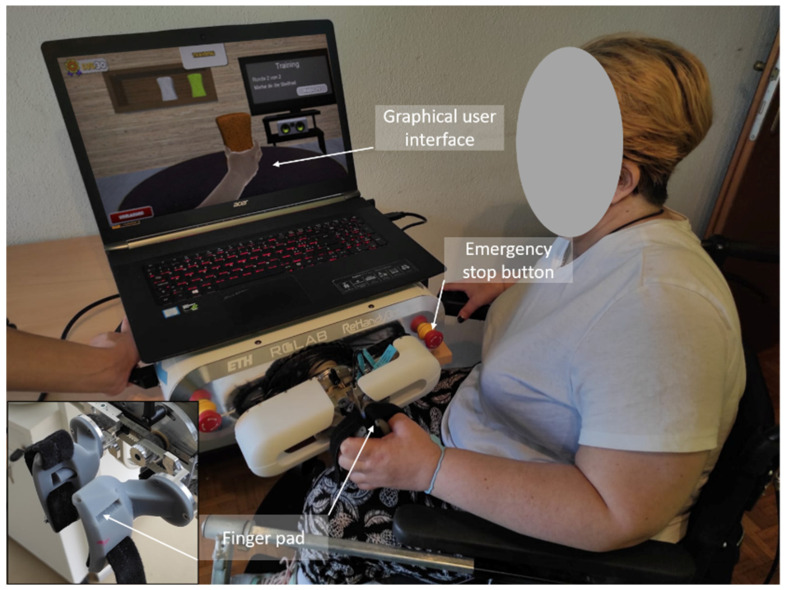
One of the study participants (subject 5) performs the hand muscle tone assessment with ReHandyBot (RHB), a haptic device including a physical (i.e., instrumented finger pads) and a graphical user interface to perform assessments and therapy exercises. During the muscle tone assessment, the subject has to relax their hand while the robot applies six 150 ms hand opening perturbations of 20 mm amplitude.

Subject two was injected with 305 U of onabotulinumtoxinA, while the others were injected with 242.5 (185) U of incobotulinumtoxinA. Contrarily to the other subjects, subjects four and five did not receive any injections in the finger muscles (i.e., flexor superficialis finger II-III-IV, lumbricales fingers II-V, flexor pollicis longus, and opponens pollicis). The personalization of the BoNT-A injection dose and site may represent a minor confounding factor for the study results but was necessary to allow for optimal tailoring of the muscle relaxation effect based on the subject’s need. The subjects presented a baseline total MAS of 12.2 (8.2) at S0, which decreased to 7.4 (5.1) at S1. In particular, the MAS in finger flexors, which mostly affect the use of the RHO, was 1.8 (1.3) at S0 and 1.2 (1.1) at S1 (i.e., a decrease of 0.6 (0.9) between S0 and S1). Peak forces assessed with a previously validated robot-assisted assessment of muscle tone using the ReHandyBot (RHB, Figure 5) (i.e., peak force at fingertip after a 20 mm ramp-and-hold 150 ms perturbation) were 11.6 (4.9) N at S0 and 9.4 (4.9) N at S1 (i.e., decrease of 2.2 (2.2) N between S0 and S1). A summary of the BoNT-A doses injected at S0 and of the MAS results at S0 and S1 are provided in Table 2 and Table 3. The MAS of the finger flexors at S0 and S1 correlates linearly (R^2^ = 0.795) with the respective peak forces measured by RHB at the same time points. The linear fit has a slope of 0.22 MAS points/N and an intercept of −0.82 MAS points. Based on the RHB peak force results, the fit predicts the MAS score of the study participants with an RMSE of 0.51 MAS points. Excluding one outlier data point, the line fit presents the same slope of the data collected in a previous study on another population suffering from chronic upper-limb spasticity after stroke [25].

### 2.5. Usability

The usability results for all the questionnaires are summarized in Table 4. Subjects ranked the RELab tenoexo system (i.e., RHO, mobile phone application) as OK (51.0 (18.8) out of 100) on the System Usability Scale (SUS). On the raw Task Load Index (rawTLX), the average workloads required to use the system were all above 50%, excluding the temporal workload, indicating that the pace at which the subjects were asked to perform the assessment/therapy tasks was not stressful for them but that the system was too complex for independent use. The mobile phone application was ranked 16.3 (3.1) out of 21 on the mHealth App Usability Questionnaire (MAUQ), with the best results in the categories ease of use (6.0 (1.4) out of 7) and usefulness (5.6 (1.3) out of 7). The RHO was ranked 3.2 (0.3) out of 5 on the Quebec User Evaluation of Satisfaction with Assistive Technology 2.0 (QUEST 2.0) (device subscore), achieving the best results in the items safety (4.0 (1.0) out of 5) and ease of use (4.4 (0.9) out of 5). Four out of five subjects stated that they could use all the functions of the RELab tenoexo and the therapy application without problems (U1). All the subjects would recommend the adjunct BoNT-A and RELab tenoexo use to other people with an impairment similar to theirs. Two out of three subjects that performed the simulated unsupervised therapy session found the therapy exercises motivating (U3). These subjects found their comfort level during the execution of the therapy exercises as 3.7 (2.3) out of 10 (U4).

During the study, the RELab tenoexo exhibited technical deficiencies affecting its usability, including fragility of Bowden cable transmission and 3D-printed thumb mechanism, inappropriate finger flexion–extension range of motion, instability in finger fixations, and disengagement of the palm transmission mechanism.

## 3. Discussion

This paper investigated the safety, feasibility, functional benefit, and usability of a novel adjunct therapy approach combining BoNT-A injections with RHO-assisted hand therapy for subjects with upper-limb spasticity after CNS lesions. The approach was evaluated in a rigorous preliminary study to evaluate its potential for long-term application without supervision. This approach promises to be a suitable solution to increase the therapy dose offered to subjects after CNS lesions in unsupervised environments (e.g., at home), with the potential to maximize and maintain long-term therapy outcomes (e.g., reduction of muscle hypertonia, improvement in functional ability levels and subject independence) and/or to reduce the frequency/dose of invasive BoNT-A injections over time. The study further allowed us to identify necessary modifications to increase the feasibility of this therapy approach in a real-world scenario.

### 3.1. The Adjunct Use of BoNT-A Injections and RELab Tenoexo Is Safe and Feasible

We proposed the use of a sleek and lightweight RHO, the RELab tenoexo [30], and a newly developed functional mobile phone therapy application that could be used already upon first exposure by spastic subjects after CNS lesions in two sessions with minimal external supervision. BoNT-A injections reduced subjects’ muscle tone without adverse events during their administration nor side effects and are therefore considered safe and feasible.

Once donned, the RELab tenoexo could be easily controlled independently by the subjects through the mobile phone application to perform both assessment and therapy tasks. During the simulated unsupervised therapy session, the subjects could independently perform all three therapy exercises with limited external assistance and small issues explained by their impairment (i.e., subjects with higher MAS or lower ARAT, such as subject five, performed worse in exercises with greater difficulty, such as the Painting exercise).

The adjunct use of the RELab tenoexo after BoNT-A injections was, in this study, safe and partially feasible due to the limited robustness of the RHO prototype. For two subjects, the RHO prototype did not last to complete the simulated unsupervised therapy session at S1 due to the fragility of the Bowden cable transmission at the level of the actuation unit. Moreover, external support from an experienced person was required by the subjects to repair minor technical failures (e.g., fragility of Bowden cable transmission and 3D-printed thumb mechanism), don and doff the hand module, or adjust its placement on the subject’s hand. The limited usability of the RELab tenoexo does not match previous successful usability evaluations in other user groups (e.g., SCI [30,31,32]). This result is not surprising, since the RELab tenoexo is a research prototype under development and has been designed primarily for subjects with hand weakness, not with spasticity, which sets higher mechanical constraints. Moreover, the hand modules used in this study were in two fixed dimensions. Improving the robustness of the RHO and tailoring the hand modules to each subject would likely positively affect the usability scores.

Nevertheless, the approach of combining an RHO and BoNT-A injections proved to be feasible, and the study presented in this paper is one of the very few successful attempts of using an RHO for subjects with upper-limb spasticity [33,34]. In fact, using an RHO for subjects with hand spasticity is challenging, and according to a recent review [35], less than 13% of RHOs in research or on the market target spastic users, typically only for muscle tone assessments [36,37]. In general, spastic users are often neglected in clinical studies on robot-assisted rehabilitation since spasticity compromises the wearability of exoskeletal systems and requires high and well-controlled forces to mobilize the joints while respecting safety (e.g., avoiding overstretching of muscles and tendons). At the same time, spasticity is frequent after CNS lesions, with an incidence above 40% after stroke and above 80% in multiple sclerosis [3,8]. In this sense, the RELab tenoexo, after minor design adjustments, is an ideal candidate for this application as it achieves sufficiently high forces while maintaining intrinsic compliance to protect the user from hand injuries.

### 3.2. The Adjunct Use of BoNT-A Injections and RELab Tenoexo Provides Functional Benefits

Adjunct RELab tenoexo therapy after BoNT-A injections provided, at first exposure, functional benefits in terms of BBT and ARAT to the subjects with hand hypertonia (i.e., MAS ≥ 1 in finger flexors). The benefits were perceived by these subjects and were greater, on average, than the single use of BoNT-A injections or RELab tenoexo alone. This finding is highly promising since, in this study, only one therapy session with the RELab tenoexo was performed after BoNT-A injections. The functional benefit may further increase as users become accustomed to the support of the RHO over multiple therapy sessions.

Two extensive studies with chronic stroke patients did not find short- or long-term additive effects of adjunct upper-limb rehabilitation on upper-limb function compared to BoNT-A alone [38,39,40]. One of them, the InTENSE trial, a large randomized controlled trial, compared BoNT-A treatment plus evidence-based movement training with BoNT-A in combination with a catalog of exercises, using the Goal Attainment Scale and BBT as primary outcomes at three months. No significant differences were found between the groups. However, increased strength in the intervention group raised doubts about whether the chosen functional scales were appropriate for capturing improvements. The intervention group practiced 62–87% of the prescribed 60 min per day despite minimal noticeable improvements, suggesting that a predefined therapy plan, like the one we propose in our study, might sufficiently motivate independent home training. The InTENSE trial suggested that less-chronic patients with severe pain and moderate impairment might benefit from the adjunct approach [38]. Compared to the longer InTENSE trial, the small functional gains in our two-session study are promising and might increase with more intensive therapy. Based on these assumptions and other promising results from similar studies [18,19,20], further investigation into the efficacy of our approach in a larger trial seems warranted.

The items that showed higher benefit in our study were the tasks requiring the manipulation of objects sized between 2.5 and 5 cm. Objects of smaller sizes cannot be grasped because the current design of the RELab tenoexo does not allow full finger flexion into a pinch grasp nor selective fine movements of individual fingers. Objects of larger size cannot be grasped either since spastic subjects cannot fully open their hands, even with RHO assistance (e.g., due to biomechanical changes in the hand, residual spasticity, weakness). This is in line with previous studies on spinal cord injury users and one user with stroke, which also identified similar functional benefits with these object dimensions and, in particular, in the grasp and grip subscores of the ARAT [30,32]. As in our study, they found that the subscores “pinch” decreased for most participants, the “gross movement” scores remained unchanged, and the subject with higher ARAT had no benefit or worsening from the use of the RELab tenoexo. Subject four in our study did not achieve functional benefits since he had a high ARAT and good hand function without finger flexor spasticity at the baseline.

The range of functional benefits measured in this study was probably compromised by the severe proximal upper limb impairment of all the subjects, in particular at the level of the shoulder. BoNT-A injections can improve shoulder function and reduce pain in post-stroke spasticity [41]. However, higher immediate functional benefits could be expected if the shoulder was supported by a powered shoulder exoskeleton or a passive gravity compensation system [42]. Compensating for gravity unloads shoulder abductors, and increases the elbow’s range of motion and the reachable workspace in subjects suffering from abnormal flexor synergy activations, which frequently accompany upper-limb spasticity [43,44,45,46].

### 3.3. BoNT-A Injections Effectively Reduce Muscle Tone

The BoNT-A injections performed in the study successfully reduced the muscle tone in upper limb muscles (i.e., flexors and extensors of elbow, wrist, fingers, and pronosupinators of the forearm) by approximately five MAS points on average, without adverse events. As pointed out by a recent metanalysis on the effectiveness of BoNT-A injections for upper-limb spasticity [47], BoNT-A injections effectively reduce resistance to passive movement (as measured by the MAS), involuntary movements, spasticity-related pain, and caregiver burden and improve passive range of motion. They have no long-term effects on arm–hand capacity, and it is still debated whether they have an effect or even negatively affect active hand and arm function. Our results showed immediate small functional gains from the RELab tenoexo in the window of reduced muscle tone opened by the BoNT-A injections and are, therefore, a promising proof of concept that an RHO can provide functional support after BoNT-A injections and improve active function, particularly over longer therapy periods.

The muscle tone of finger flexors was reduced by 0.6 MAS points and 2.2 N in the RHB assessment. The tone measurements via the MAS and the RHB showed a higher correlation in this study and a similar linear relationship compared to previous investigations [25]. The advantage of a robotic assessment such as the one with RHB is that the robot could automatically monitor muscle tone changes without the presence of a therapist and guarantee objective measurements during well-controlled and reproducible assessment conditions (i.e., velocity and force measurement). This is particularly useful in view of future home therapy applications. Moreover, the robotic assessment is more accurate compared to the MAS, which has limited resolution and reliability [25,48,49].

### 3.4. The RELab Tenoexo System Showed Positive Usability in a Challenging Population

It is highly satisfactory that an early-stage prototype, such as the RELab tenoexo system, achieved pilot usability results between intermediate and good despite the challenging (spastic) patient population tested. This result is very promising and underlines the need to further evaluate this therapy approach (i.e., its efficacy) in a larger clinical trial. The system had an average “OK” SUS score of approximately 50 out of 100 [50] and an intermediate perceived workload of around 50% on the rawTLX. Such results are similar to those of other RHOs, which showed “OK” to “best imaginable” scores at the SUS [51,52,53] and workloads between 10% and 90% at the rawTLX [51,54,55]. The QUEST 2.0 score of the RHO was about 3 out of 5. The HERO glove had a comparable QUEST 2.0 result of 3.3 [42]. Considering that lower-limb exoskeletons and home assistance robots rated between 3 and 4 on QUEST 2.0 are widely used for rehabilitation and assistance, our result is considered positive [56,57]. The mobile phone application used for intention detection and therapy was highly rated on the MAUQ, with a total score of approximately 16 out of 21 and subscores of about 6 out of 7 in ease of use, interface and satisfaction, and usefulness, outperforming other healthcare apps typically scoring between 3 and 4 [58,59]. Usability evaluations using SUS, TLX, and QUEST 2.0 are common in technology-assisted rehabilitation [60], but directly comparing different devices and studies remains difficult due to varying applications, user groups, environments, and small sample sizes [61].

It should be noted that the three subjects with higher impairment in terms of both finger flexors’ tone (i.e., MAS between 1 and 3) and overall upper-limb function (i.e., ARAT between 3 and 5) gave lower ratings on the SUS and rawTLX. Subject five also gave low ratings on the QUEST 2.0 and MAUQ. This information, and the technical limitations that the RELab tenoexo showed during the sessions, are key findings of this pilot study. First, these findings underline weak points in the design of the RELab tenoexo that should be carefully addressed to meet the needs of severely impaired patients before starting a larger clinical trial focused on efficacy. Second, they can be beneficial in clarifying which target population could benefit from the adjunct BoNT-A and RHO therapy approach (see Section 3.5).

### 3.5. Limitations and Future Work

Our preliminary results should be interpreted with respect to the small sample size tested. However, this size can be considered sufficient to pilot-assess the safety and feasibility of the approach and to identify major usability challenges at an early stage [62]. The results should be further validated over a longer time horizon (with multiple RHO-assisted therapy sessions) in a larger population, either in real unsupervised conditions (e.g., in the home environment) or in supervised (or group) therapy settings, where therapists could assist subjects in donning and doffing. This could allow for verification of the feasibility and efficacy of this therapy approach (i.e., on muscle tone and upper-limb function), whether subjects could learn to use the RHO independently and progressively improve their performance, and if this would lead to a reduced need of BoNT-A injections (i.e., a reduction in injections’ dosage or frequency). Finally, since this study did not focus on efficacy, the medical doctors and assessors involved in the study were not blinded to the treatment modality and the scope of the study.

Before starting a larger clinical trial on efficacy, the RELab tenoexo prototype should undergo a minor redesign to increase its robustness since, at the moment, an experienced person is always needed to assist the subjects in donning and doffing the hand module and in fixing technical issues. The technology readiness level (TRL, ref. [63]) of the RELab tenoexo is currently approximately level six for the target application presented in this study and should be brought to at least level seven by improving mainly the Bowden cable transmission, the finger fixations, and the range of motion. The results of this study are a promising basis to shape the inclusion/exclusion criteria for the target population of such a study. The adjunct BoNT-A and RHO therapy approach could be primarily beneficial for subjects after CNS lesions with moderate monolateral impairment (i.e., ARAT between 20 and 38, ref. [64]), hand hypertonia (i.e., MAS between 1 and 3), and no major cognitive or visual impairment affecting their ability to use a mobile phone therapy application. Subjects with severely impaired shoulder function or range of motion (e.g., due to weakness, contracture, spasticity) should be given the possibility to train using arm/shoulder gravity supports (e.g., SaeboMAS mini [65], Myoshirt [66]). Carefully establishing optimal timing for assessments, adjunct hand therapy and BoNT-A treatments will be necessary to improve motor function and spasticity [19].

## 4. Materials and Methods

### 4.1. RELab Tenoexo and Mobile Phone Application

**Figure 6:**
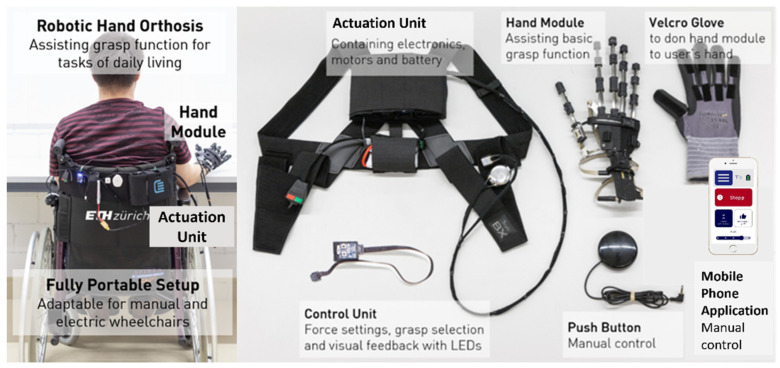
RELab tenoexo overview: the RELab tenoexo can be worn by the user or mounted on a wheelchair (**left**). The hand module (148 g) can be placed on a user’s hand using a glove with Velcro and button-clips and is actuated by motors placed in the actuation unit (720 g) together with the electronics. Different intention detection strategies (e.g., push button, mobile phone application) can be used to trigger the motion of the RHO (**right**).

**Figure 7:**
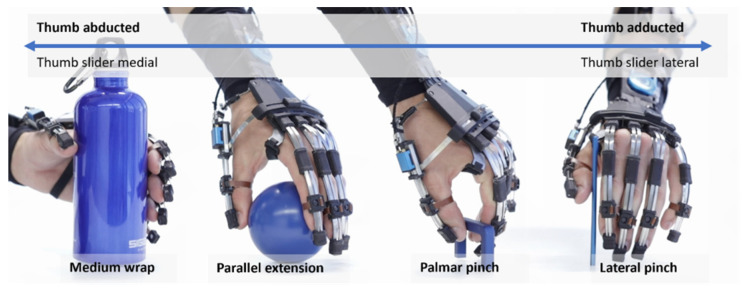
Grasp functions of the RELab tenoexo (adapted from [31]): the compliant structure of the finger mechanism allows for adapting to the shape of different objects. Four grasp types (denomination according to [71]) can be executed with the RELab tenoexo by moving the thumb slider at the back of the hand module from medial to lateral.

The proposed study utilizes the RELab tenoexo, a fully wearable RHO, designed for assisting individuals with sensorimotor hand impairments during daily activities [30,31]. The RHO includes a lightweight hand module and an actuation unit (i.e., actuation system, electronics, and battery) (Figure 6), connected via Bowden cables. The hand module’s spring-blade mechanism [30,67] assists finger flexion and extension biomimetically, allowing for a slim design. It supports four common grasp types (i.e., medium wrap, parallel extension, palmar pinch, and lateral pinch) using two motors (Figure 7), one for the fingers and one for the thumb. The remote actuation reduces hand/arm load to only 150 g, enhancing portability and ergonomic use [68,69,70]. Case studies in SCI patients showed immediate improvements in upper-limb function and reduced compensatory movements [30].

**Figure 8:**
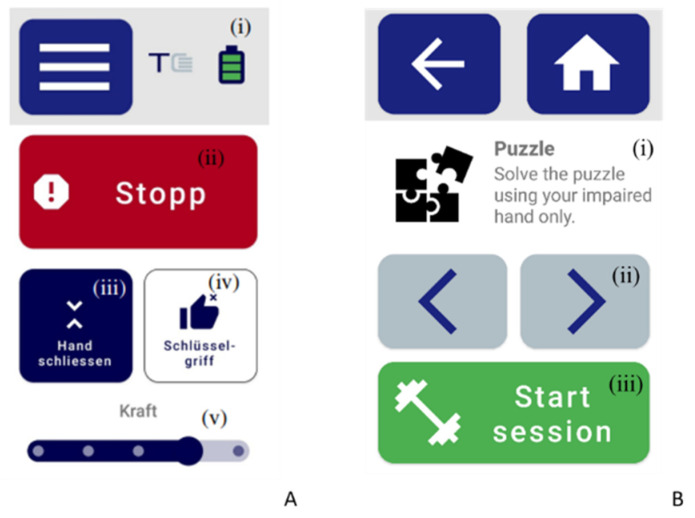
RELab tenoexo mobile phone application. (**A**) Main screen offering five functions: (**i**) battery information, (**ii**) emergency stop, (**iii**) opening and closing of the hand, (**iv**) changing of the grip type (i.e., cylindrical grip, key grip), and (**v**) force selection. (**B**) Therapy screen: the user can navigate between different therapy exercises (represented with an icon and a short description (**i**)) using left/right arrows (**ii**) and start the exercise with the green button (**iii**). During the exercise, the application records end-point accelerations from an XSens DOT accelerometer, which can be attached to the dorsum of the RELab tenoexo, but this feature was not used for the scope of this study.

Various intention detection options (e.g., sensors/devices to detect user input commands) enable the RHO to trigger hand opening and closing based on user intention. For this study, a mobile phone application was developed as the primary intention detection strategy to control the RHO and facilitate therapy exercises. This application was created with input from an experienced physiotherapist and an engineer using an iterative user-centered design approach involving three users with chronic stroke [68]. To support future use in unsupervised settings such as home environments, the application allows users to autonomously control hand opening and closing, grip type, and maximum force (Figure 8A). Additionally, the therapy window (Figure 8B) enables users to select and initiate therapy exercises. During exercises, the application records data on exercise type, number of hand opening and closing repetitions, total therapy time, and emergency states.

**Figure 9:**
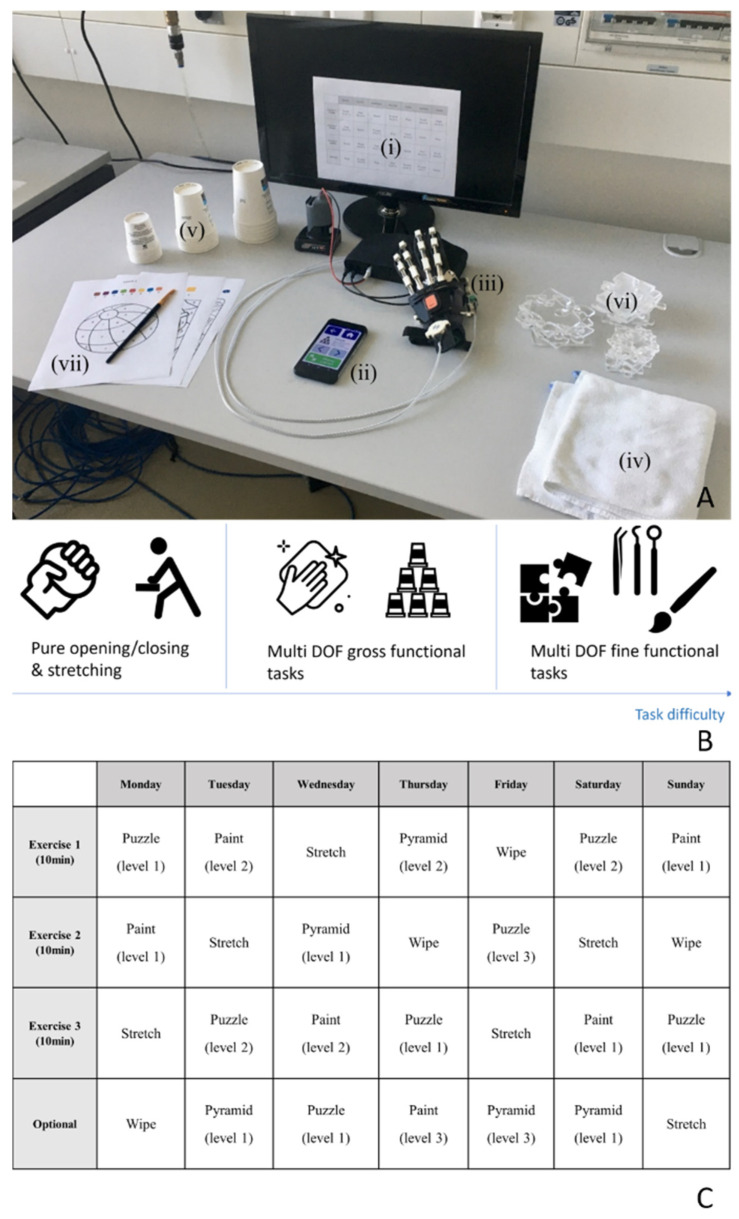
Example of therapy setup with the RELab tenoexo. (**A**) Based on a predefined therapy plan (i), the user can use the mobile phone application (ii) to perform a set of therapy exercises with the RELab tenoexo (iii). (i.e., Cleaning (iv), Pyramid (v)). Mildly impaired subjects will perform fine functional tasks (i.e., Puzzle (vi), Painting (vii)). (**B**) The therapy exercises have increasing difficulty, depending on the subject’s ability level. Severely impaired subjects will perform hand opening–closing motor training or the Stretching exercise. Moderately impaired subjects will perform gross functional tasks requiring the recruitment of hand and proximal upper limb joints. (**C**) Tabular therapy plan used in the study. Participants identified “Wednesday” and performed the three 10 min mandatory exercises: Stretching, Pyramid (difficulty level 1: glasses of 3 cm), and Painting (difficulty level 2).

The therapy exercises are inspired by daily life activities. Based on the patient’s ability level, a therapist can create a weekly therapy protocol including exercises of varying difficulties, as shown in Figure 9. The exercises include:

1. Stretching: Extend fingers on the table surface by lying on the hands using own body weight and the assistance of RELab tenoexo, repeated ten times for one minute each.
2. Cleaning: Clean a surface with a towel using the RELab tenoexo, with ten one-minute sessions and ten-second breaks to refold the towel.
3. Pyramid: Build and disassemble a pyramid with plastic glasses within ten minutes, adjusting difficulty with glass diameters (three to six cm).
4. Puzzle: Complete a puzzle within ten minutes, with difficulty based on puzzle piece sizes (four to ten cm).
5. Painting: Paint numbered cells on a paint-by-number sheet within ten minutes, with varying cell sizes affecting difficulty (Figure 2).

### 4.2. Pilot Study Protocol

We conducted a pilot study involving five subjects with spasticity in the upper limb muscles due to a chronic lesion in the CNS (>6 months). The participants provided written informed consent, and the study was approved by the ETH Zurich Ethics Commission, Switzerland (EK 2021-N-32) after a declaration of non-responsibility from the Cantonal Ethics Committee of Zurich (req-2022-01509). Subjects were recruited at the EOC Neurocentro—Istituto di Neuroscienze Cliniche della Svizzera Italiana in Lugano, Switzerland, where the study was conducted. Inclusion criteria were as follows:

- Age between 18 and 90 years;
- Chronic stroke or other lesions to the CNS (>6 months from onset);
- National Institutes of Health Stroke Scale (NIHSS) ≥ 1 in at least one item regarding upper-limb motor function, sensory functions, or ataxia;
- Modified Ashworth Scale (MAS, ref. [72]) ≥ 1 in one or more of the following muscles: forearm pronator and supinator, flexors and extensors of elbow, wrist, and fingers;
- Ability to read, understand, and sign the informed consent.

Exclusion criteria were:

- Moderate to severe aphasia: Goodglass–Kaplan scale < 3;
- Moderate to severe cognitive deficits: levels of cognitive functioning-revised (LCF-R) < 8;
- Functional impairment of the contralateral limb;
- MAS = 4 in flexors or extensors of wrist or fingers;
- Severe pain in the affected arm: visual analogue scale for pain (VASp) ≥ 5;
- Other pathologies that may interfere with the study.

**Figure 10:**
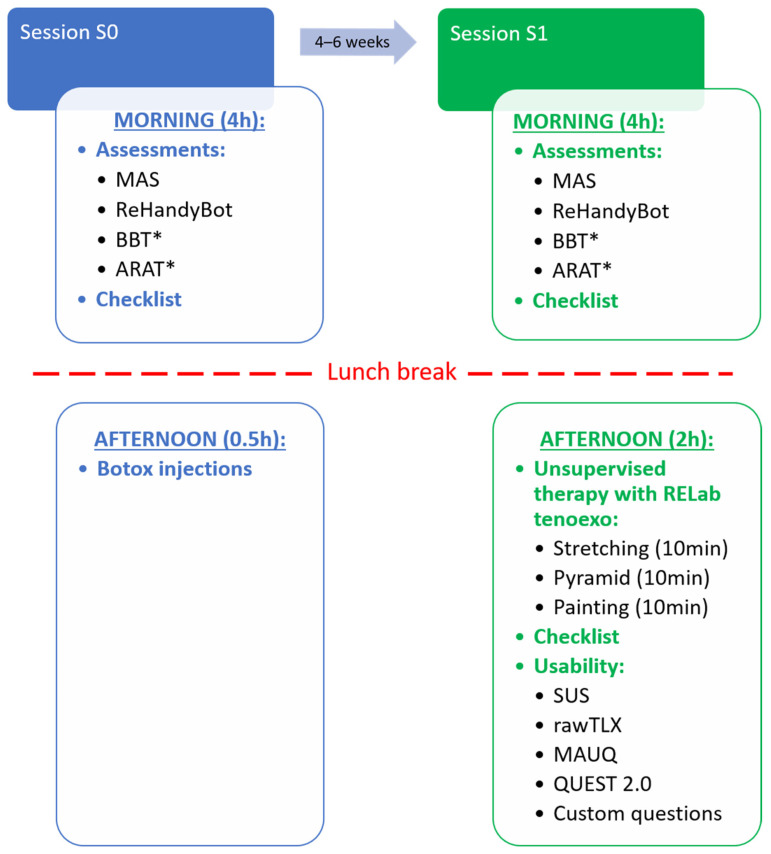
The pilot study consisted of two sessions, S0 and S1. In the morning of S0, subjects performed baseline assessments while the experimenter evaluated whether the subject could autonomously don/doff and control the RELab tenoexo using the mobile phone application. In the afternoon, subjects received BoNT-A injections. After four to six weeks (session S1), when the BoNT-A effect peaks, the subjects repeated the assessments. In the afternoon, they performed a simulated unsupervised therapy session with the RELab tenoexo consisting of three 10 min exercises (Stretching, Pyramid, and Painting) performed with the mobile phone application while the experimenter recorded which tasks the subject could not perform autonomously. At the end of the study, the subjects answered usability questionnaires. * = assessment performed with and without the RELab tenoexo. Abbreviations: MAS = Modified Ashworth Scale, BBT = Box and Block Test, SUS = System Usability Scale, rawTLX = raw Task Load Index, MAUQ = mHealth App Usability Questionnaire, QUEST 2.0 = Quebec User Evaluation of Satisfaction with Assistive Technology 2.0.

The study protocol, detailed in Figure 10, included two sessions (S0 and S1), each with a morning and afternoon part. In S0, subjects underwent conventional and robotic assessments to determine their baseline ability level (see Section 4.3), learned to use and control the RELab tenoexo via a mobile app, and then repeated the assessments with the device. In the afternoon, subjects received BoNT-A injections by an experienced medical doctor in spastic upper limb muscles, under electromyographical guidance (details on injected muscles and BoNT-A dose are in Table 2). Injected doses ranged from 30 to 450 U of incobotulinumtoxinA (Xeomin^®^) or onabotulinumtoxinA (Botox^®^), both Class A, recommended by the American Academy of Neurology Practice Guidelines [73]. Injection specifics (e.g., muscle, BoNT-A preparation, dose) were personalized based on the participant’s goals, medical history, and previous BoNT-A effects.

After four to six weeks (S1), when BoNT-A effects peaked, subjects repeated the assessments with and without the RELab tenoexo. In the afternoon, they were briefly introduced to therapy exercises and performed a simulated unsupervised therapy session with the RELab tenoexo and the therapy application. They followed a predefined therapy plan (Figure 9C), performing three mandatory 10 min exercises: Stretching, Pyramid (difficulty level 1: 3 cm glasses), and Painting (difficulty level 2) (see Section 4.1). An investigator, intervening only in case of risk or upon request, observed and recorded on a checklist which tasks the subjects could or could not perform independently (see Figure 1). Participants used their impaired hand for all assessments and exercises.

### 4.3. Outcome Measures

The primary outcome of the study was the safety and feasibility of adjunct BoNT-A injections and simulated unsupervised therapy with the RELab tenoexo. To assess safety, we recorded adverse events and conditions that could put the safety of the subject at risk, related to the study intervention (i.e., BoNT-A injections or RELab tenoexo system). Feasibility was evaluated through a performance checklist filled out by the investigator while observing the participants in sessions S0 and S1. The checklist recorded which items (tasks and actions) the subject could or could not perform without supervision or in which the investigator’s help was required.

The checklist includes 21 items described in Figure 1. Four items “DN” are about donning the RHO. Four items “CO” are about starting up the RHO (e.g., the power, motors, and mobile phone application) and the ability to manually control the opening and closing of the RHO through the mobile phone application. Ten items are about the execution of the simulated unsupervised therapy session: three items “TH” are about the ability to start any exercise and grasp/release objects used in the exercise, one item “ST” is specific for the Stretching exercise, two “PY” are specific for the Pyramid exercise, and four items “PA” are specific for the Painting exercise. Three items “DF” are about turning off and doffing the RHO. The items “DN”, “CO”, and “DF” were evaluated during the assessments at S0 and S1. The therapy-related items were evaluated during the simulated unsupervised therapy session at S1. The results of the checklist per subject are calculated as the percentage of items that required intervention with respect to the total performed items.

There are three secondary outcomes of the study:

1. Functional Benefit: The following assessments and questions were used to evaluate, compared to the baseline, the immediate functional benefit of the RELab tenoexo alone at S0 as well as the functional benefits of the BoNT-A injections and the adjunct use of RELab tenoexo after BoNT-A injections at S1. Gross manual dexterity was evaluated with the BBT [74]. Upper-extremity performance (i.e., coordination, dexterity, and functioning) was assessed with the ARAT [75]. At the end of session S1, subjects were asked the question: “Q1: Did you perceive an immediate functional benefit when wearing the RELab tenoexo compared to without wearing it?”
2. Assessment of Muscle Tone: The MAS was used to assess at the baseline (S0) and after BoNT-A injections (S1) the tone level of the flexor and extensor muscles of the elbow, wrist, finger, and pronosupinator muscles of the forearm. The MAS was chosen as it is the most widely used test for the measurement of muscle hypertonia and spasticity in research and clinical practice [76]. In parallel, the muscle tone in finger flexors was also assessed using a robotic ramp-and-hold perturbation method developed by the author with the hand rehabilitation robot RHB (Figure 5), which can more accurately capture the level of muscle tone (for more details, please refer to [25]). The relationship between the RHB measurement (i.e., average force reaction at the fingertips after six 150 ms ramp-and-hold hand opening perturbations of 20 mm) and the MAS score is then verified.
3. Usability: The usability of the RELab tenoexo system (i.e., RHO and mobile phone application) was evaluated using standard and custom questionnaires administered at the end of the study (S1). The overall usability of the system and the workload perceived by the user while using it were assessed through the SUS [77] and the rawTLX [78], respectively. The specific usability of the RHO and of the mobile phone application were evaluated using the QUEST 2.0 [79] and the MAUQ [80], respectively. The following custom questions were asked to the users at the end of the study (S1): “U1: Could you use all the functions of the RELab tenoexo and the therapy application without problems?”, “U2: Would you recommend the adjunct BoNT-A and RELab tenoexo therapy approach to other people with an impairment similar to yours?”, “U3: Were the therapy exercises motivating?”, and “U4: How comfortable was the execution of the therapy exercises for you between 1 (not comfortable at all) and 10 (extremely comfortable)?”

The BBT and ARAT are widely used to assess upper-limb function according to the International Classification of Functioning, while the MAS evaluates body function and structures passively [81]. In the BBT, subjects move 2.5 cm wooden blocks between two compartments within 60 s, with scores ranging from 0 (severely impaired) to 61–70 blocks (healthy men aged 60+) [74]. The ARAT consists of 19 items across four subsections (i.e., grasp, grip, pinch, and gross arm movement) [75], rated on a 4-point scale from 0 (cannot perform) to 3 (performs normally), with total scores from 0 to 57, indicating overall upper-limb functional performance (i.e., 0–19 low, 20–38 moderate, and 39–57 high) [64].

MAS and RHB assessments were used to determine muscle tone at S0 and S1. MAS rates resistance to passive stretch on a 6-point scale from 0 (no increase) to 4 (rigid), reported as 0–5 to account for the 1+ in the scale. The RHB, a haptic device, was used to assess hand muscle tone through six fast (150 ms) 20 mm ramp-and-hold perturbations to extend the finger flexors while the subject was at rest [25]. The average peak force at the finger pads was used to compute tone. The small perturbation amplitude (10 mm at each fingertip) prevents overstretching and reduces perceptibility, as awareness changes can influence muscle tone [82,83].

The SUS, the rawTLX, and the QUEST 2.0 can be used to measure the usability of wearable robotic devices [60]. The SUS is a ten-item questionnaire rating overall usability on a scale from 0 to 100, with scores above 50 indicating acceptable usability [50,77]. The rawTLX assesses workload across six domains: mental, physical, temporal demands, performance, effort, and frustration, ideally scoring below 50% [78]. The QUEST 2.0 measures satisfaction with assistive technology across eight device-related items, with scores above 3 out of 5 indicating satisfaction levels between “more or less satisfied” and “very satisfied” [79].

The MAUQ evaluates the usability of health-related mobile applications in clinical settings, assessing ease of use, interface satisfaction, and usefulness on a 7-point Likert scale, with higher scores indicating better usability [80].

### 4.4. Data Analysis

Assessment results, answers from questionnaires, and population results of the checklist were analyzed via descriptive statistics and are reported as means with standard deviations (i.e., mean (std)), to represent the average tendency and spread in subjects’ characteristics/responses, respectively. For better readability and interpretation, the feasibility results (i.e., the checklist) are presented graphically as a heat map, while the assessment results are presented as box plots. Such simple statistics were selected because of the very small sample size, which does not safely allow for assessment of the normality of the data and may include a disproportionately small or large number of outliers that would significantly skew the results of statistical tests [84]. The coefficient of determination R^2^ and the root mean square error (RMSE) were used to estimate, respectively, the proportion of the variation and the error in the MAS score of finger flexors that is predictable from the RHB peak forces through a linear regression fit. All the analyses were performed using Microsoft Excel (version 2407).

## Figures and Tables

**Table 1:** Baseline characteristics. Abbreviations: NIHSS = National Institutes of Health Stroke Scale, LCF-R = Levels of Cognitive Functioning Revised, VASp = Visual Analogue Scale for pain, ARAT = Action Research Arm Test, MAS = Modified Ashworth Scale, MAS total = sum of MAS scores of flexors and extensors muscles of the elbow, wrist, fingers, and pronosupinators muscles of the forearm.

Category (Range)	Sbj 1	Sbj 2	Sbj 3	Sbj 4	Sbj 5	Mean (Std)
Gender (male, female)	F	M	M	M	F	3 M, 2 F
Age (years)	54	59	48	82	49	59.5 (13.7)
Hand dominance (left, right)	R	R	R	R	R	5 R
Impaired hand (left, right)	R	L	R	R	L	2 L, 3 R
Lesion type	ischemic stroke left MCA	ischemic stroke right MCA	ischemic stroke left ACA and MCA	occlusive dissection of the left ICA	right vestibular Schwannoma resection	-
Time post-lesion (years)	11.3	15	4.7	23.2	2.8	11.4 (7.4)
NIHSS motor arm (4)	0	1	0	1	2	0.8 (0.8)
NIHSS sensory (2)	0	2	1	1	0	0.8 (0.8)
NIHSS ataxia (3)	0	0	0	0	2	0.4 (0.8)
Goodglass–Kaplan (5)	5	5	3	5	3	4.2 (1.0)
LCF-R (10)	10	10	9	10	10	9.8 (0.4)
VASp (10)	0	0	3	0	0	0.6 (1.2)
ARAT (57)	25	3	5	23	3	11.8 (10.0)
MAS total (40)	19	23	5	6	8	12.2 (8.2)
MAS finger flexors (5)	3	3	1	0	2	1.8 (1.3)

**Table 2:** Dose of BoNTAs (units) injected in upper-limb muscles with EMG guidance. Abbreviations: IBT: incobotulinumtoxinA; OBT: onabotulinumtoxinA.

	Sbj 1	Sbj 2	Sbj 3	Sbj 4	Sbj 5
Botulinum toxin	OBT (Botox^®^)	IBT (Xeomin^®^)	IBT (Xeomin^®^)	IBT (Xeomin^®^)	IBT (Xeomin^®^)
Posterior deltoid	-	-	-	50	-
Trapezius horizontalis	30	-	-	-	-
Latissimus dorsi	-	-	-	60	-
Infraspinatus	-	-	-	60	-
Teres major	50	-	-	60	-
Pectoralis major	30	35	-	50	-
Biceps brachii	50	30	-	-	-
Brachialis	40	60	-	50	-
Brachioradialis	50	30	-	-	-
Flexor carpi radialis	20	40	50	-	30
Pronator teres	40	40	30	-	-
Flexor superficialis finger II-III-IV	100	-	60	-	-
Lumbricales fingers II-V	-	30	-	-	-
Flexor pollicis longus	-	20	20	-	-
Opponens pollicis	-	20	-	-	-

**Table 3:** Modified Ashworth Scale (MAS) and ReHandyBot assessment (i.e., peak force at fingertip after a 20 mm ramp-and-hold 150 ms perturbation) results at S0 and S1.

			S0					S1		
	Sbj 1	Sbj 2	Sbj 3	Sbj 4	Sbj 5	Sbj 1	Sbj 2	Sbj 3	Sbj 4	Sbj 5
Elbow flexors	3	5	1	2	1	2	5	1	1	1
Elbow extensors	4	1	1	0	2	3	0	0	0	1
Forearm pronators	3	5	0	1	0	2	4	1	0	1
Forearm supinators	1	2	0	0	2	0	0	0	0	0
Wrist flexors	3	4	2	3	1	0	3	2	2	1
Wrist extensors	2	3	0	0	0	0	1	0	0	0
Finger flexors	3	3	1	0	2	1	3	1	0	1
Finger extensors	0	0	0	0	0	0	0	0	0	0
**MAS total**	**19**	**23**	**5**	**6**	**8**	**8**	**16**	**5**	**3**	**5**
**Mean peak force RHB [N]**	**12.2**	**17.7**	**10.2**	**4.4**	**13.5**	**6.4**	**15.9**	**10.3**	**3.0**	**11.6**

**Table 4:** Usability questionnaires’ results.

Questionnaire (Max Value)	Value
**System Usability Scale (SUS) (100)**	**51.0 (18.8)**
**raw Task Load Index (rawTLX)**	
Mental (%): “How mentally demanding was the task?”	54.0 (39.0)
Physical (%): “How physically demanding was the task?”	56.0 (35.6)
Temporal (%): “How hurried/rushed was the pace of the task?”	10.0 (5.0)
Performance ^1^ (%): “How successful were you in accomplishing what you were asked to do?”	56.0 (35.8)
Effort (%): “How hard did you have to work to accomplish your level of performance?”	80.0 (34.1)
Frustration (%): “How insecure, discouraged, irritated, stressed and annoyed were you?”	66.0 (44.4)
**mHealth app usability questionnaire (MAUQ) (21)**	**16.3 (3.1)**
Ease of use (7)	6.0 (1.4)
Interface and satisfaction (7)	4.7 (1.0)
Usefulness (7)	5.6 (1.3)
**Quebec User Evaluation of Satisfaction with Assistive Technology 2.0 (QUEST 2.0): Device subscore (5)**	**3.2 (0.3)**
Dimension (5): “How satisfied are you with the dimensions (size, height, length, width) of your assistive device?”	2.2 (0.8)
Weight (5): “How satisfied are you with the weight of your assistive device?”	3.6 (1.5)
Ease in adjusting (5): “How satisfied are you with the ease in adjusting the parts of your assistive device?”	2.6 (1.5)
Safety (5): “How satisfied are you with how safe and secure your assistive device is?”	4.0 (1.0)
Durability (5): “How satisfied are you with the durability (endurance) of your assistive device?”	3.0 (1.6)
Ease of use (5): “How satisfied are you with how easy it is to use your assistive device?”	4.4 (0.9)
Comfort (5): “How satisfied are you with how comfortable your assistive device is?”	3.2 (1.1)
Effectiveness (5): “How satisfied are you with how effective your assistive device is in meeting your needs?”	2.4 (1.3)
**Custom questions**	
U1: “Could you use all the functions of the RELab tenoexo and the therapy application without problems?”	4 Y, 1 N
U2: “Would you recommend the adjunct botox and RELab tenoexo therapy approach to other people with an impairment similar to yours?”	5 Y
U3: “Were the therapy exercises motivating?”	2 Y, 1 N
U4: “How comfortable was the execution of the therapy exercises for you between 1 (not at all) and 10 (absolutely)?”	3.7 (2.3)

^1^ In this question, low workload corresponds to “Perfect” and high workload to “Failure”.

## Data Availability

The raw data supporting the conclusions of this article will be made available by the authors on request.
